# Incidental Prenatal Diagnosis of Congenital Inguinal Hernia: A Case Report

**DOI:** 10.7759/cureus.54356

**Published:** 2024-02-17

**Authors:** Samir Asfour, Abdelsalam Alkharouf, Yara Sultan, Laila Qarawi, Amal Shraim, Muhannad Wael

**Affiliations:** 1 Obstetrics and Gynecology, Saint Joseph Hospital, Jerusalem, PSE; 2 Urology, Saint Joseph Hospital, Jerusalem, PSE; 3 Faculty of Medicine, An-Najah National University, Nablus, PSE

**Keywords:** prenatal ultrasound, prenatal diagnose, general obstetrics, obstetrics & gynecology, gynaecology and obstetrics

## Abstract

Prenatal congenital inguinal hernia is a rare condition, with limited cases reported in the literature. Accurate prenatal diagnosis is crucial for appropriate management and outcomes. We report a case of a 44-year-old woman at 36 weeks of gestation with well-controlled gestational diabetes diagnosed with prenatal congenital inguinal hernia. The patient’s antenatal history included abnormal first-trimester screening tests for Down syndrome, but subsequent amniocentesis revealed no chromosomal abnormalities. Ultrasonography at 36 weeks showed an enlarged right scrotum with heterogeneous consistency and visible bowel peristaltic waves without signs of bowel obstruction, strangulation, or incarceration. At 39 weeks, oligohydramnios was diagnosed, leading to a decision for labor induction. However, the patient underwent a cesarean section upon her desire, giving birth to a male infant with congenital inguinal hernia. Both mother and child had a normal six-month postpartum follow-up. This case underscores the significance of detailed third-trimester ultrasonography in diagnosing prenatal congenital inguinal hernia. Early detection allows for better planning and management, highlighting the value of routine prenatal assessments for fetal organ status and early identification of malformations.

## Introduction

The tunica vaginalis, originating from the peritoneum, descends into the scrotum and the testicles during fetal development. Typically, the inguinal canal remains open during pregnancy and closes after birth. If it fails to close, the persistent process of the vaginalis may allow intestinal loops to enter the inguinal canal due to increased intra-abdominal pressure, leading to hernia formation. Indirect inguinal hernia is the most prevalent abdominal wall defect in infants and children [1].

Although congenital inguinal hernias are fairly common in pediatric patients, prenatal diagnosis during antenatal care is exceedingly rare.

From 1991 to 2016, only 16 cases were reported in the English literature [1]. Congenital inguinal hernias are relatively common among neonates and infants, with an incidence at birth of 0.88-4.4% [2]. We present a case of prenatal congenital inguinal hernia, highlighting its ultrasonographic features and the multidisciplinary management approach in our hospital.

## Case presentation

A 44-year-old woman (G8P7A0) at 36 weeks' gestation was incidentally diagnosed with gestational diabetes during a routine antenatal visit at our hospital’s outpatient clinic. Her antenatal history was significant for abnormal first-trimester screening tests for Down syndrome, indicating a 1:5 risk on combined screening at another outpatient clinic. Subsequent to this, amniocentesis showed no chromosomal abnormalities. Prenatal ultrasound examinations revealed no abnormalities; nuchal translucency was normal at 2.6 mm.

**Figure 1:**
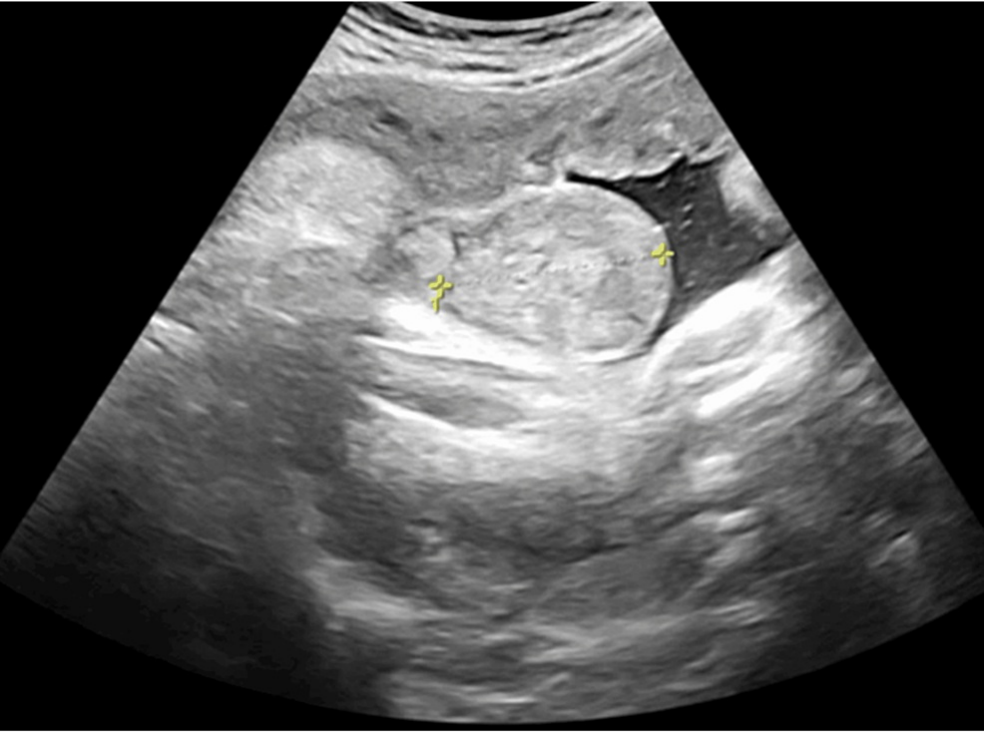
Transverse ultrasonographic image of fetal scrotum at 36 weeks gestational age, displaying right-sided enlargement (5.7 x 3.4 cm)

The patient was evaluated by our fetologist four days later. An ultrasound (Figure 1) revealed a right-sided enlarged scrotum (5.7 x 3.4 cm) with heterogeneous consistency and a mild cystic component. The ultrasound showed visible bowel peristaltic waves without vascularity and no evidence of bowel obstruction, strangulation, or incarceration.

The neonatology and pediatric departments were alerted to the patient’s condition for postnatal follow-up and potential surgical intervention. At 39 weeks of gestation, the patient returned for follow-up. Her gestational diabetes remained controlled. An ultrasound indicated oligohydramnios (the deepest vertical pocket of 2.9 cm). Consequently, labor induction using a cervical ripening agent (Prostaglandin E2) was planned. After one dose of Prostaglandin E2, the patient opted against further labor induction and underwent a cesarean section. She delivered a well-developed male baby weighing 3,140 grams, with Apgar scores of 9 at 1 minute and 9 at 5 minutes, with normal general appearance, open fontanelles, a red reflex seen bilaterally, good bilateral air entry, regular heart rhythm, and no murmurs. Abdomen was soft and lax, normal male genitalia, and inguinal hernia was seen clinically. Testes were difficult to palpate. 

Both mother and baby had an uneventful follow-up during the first six months postpartum.

As the hernia did not resolve and the mother is on a follow-up schedule for future laparoscopic hernia repair, the mother was clearly instructed about the alarming signs for urgent surgery, like abdominal distention, swelling, redness, skin changes, and irritability. 

## Discussion

Prenatal congenital inguinal hernia is an extremely rare condition. The first documented case in the English literature was reported by Meizner et al. in 1992 in Israel [3]. This report emphasizes the importance of ultrasonographic characteristics in diagnosing prenatal congenital inguinal hernia. Although pediatric inguinal hernia typically presents in isolation, it can be associated with other anomalies, such as trisomy 18, like as in the case report published by Dr. D. Paladini et al. in 1996 [54].

Targeted organ ultrasonography during the third trimester can effectively identify features of the inguinoscrotal contents documented in the literature, a case report of incidental diagnosis of fetal gallstones in the third trimester by Borza et al. [5]. 

Accurate differentiation of inguinoscrotal hernia from other scrotal masses is crucial, as management and prognosis vary significantly. Differential diagnoses include hydrocele, urogenital anomalies, testicular tumors, and teratomas.

The differential diagnosis includes other masses protruding from the abdominal wall (omphalocele) or the perineal region (sacrococcygeal teratoma, hydrocele) [4] noticing the bowel peristalsis movement extend inside the male scrotum is pathognomonic and confirms a hernia diagnosis, excluding other conditions.

Observation of peristalsis within the scrotum on real-time sonography is pathognomic for bowel herniation [6].

Fluid-filled cystic structures within the scrotum suggest bowel involvement, while solid, hyperdense contents require careful examination to rule out other diagnoses.

The most sensitive sonographic indicator of fetal inguinoscrotal hernia is the visualization of bowel peristaltic waves within the scrotum, a pathognomonic feature [4]. Clinicians must be vigilant in identifying signs of bowel incarceration and strangulation, indicated by dilated bowel loops, abnormal bowel movements, and diminished blood flow. The management of these cases is critical and lifesaving, often requiring urgent surgery instead of the elective herniorrhaphy (either laparoscopic or open, depending on the surgeon’s expertise) typically performed when the hernia is uncomplicated, non-incarcerated, and non-strangulated.

## Conclusions

Routine prenatal care, including targeted organ ultrasonography, is essential for a detailed assessment of fetal organ status and the early detection of malformations. Reporting rare findings, such as prenatal congenital inguinal hernia, during prenatal care is vital. This condition’s most significant and pathognomonic ultrasonographic feature is the visible movement of bowel peristaltic waves.
